# Meta-analysis of probiotics metabolites in gastrointestinal tract and metabolic health

**DOI:** 10.3389/fcimb.2025.1619501

**Published:** 2025-06-19

**Authors:** Xiangning Ma, Hongjun Zhang

**Affiliations:** Department of Gastroenterology, Jinan Lixia District People’s Hospital, Jinan, Shandong, China

**Keywords:** probiotic-derived metabolites, gastrointestinal tract, host-microbiota interaction, metabolite-protein network, tissue-specific gene expression, reactome pathway

## Abstract

**Introduction:**

The gastrointestinal (GI) tract acts as an essential interface between the host and the microbiota, with microbial metabolites exerting a significant role in regulating host physiology.

**Methods:**

Integrative network-based methodology that combines metabolite-protein interactions with tissue-specific transcriptomics to uncover host targets of probiotic-derived metabolites and determine their potential biological significance. Utilizing curated interaction data, it is about to construct metabolite-host protein network and prioritised genes using centrality metrics. Gene expression analysis across human tissues indicated that some high-degree genes, including SLC27A4, LCN12, and APOD, are abundant in GI areas including small intestine, colon, and duodenum, indicating a potential role in local host-microbe interactions. Further metabolite-specific expression analysis revealed separate but overlapping expression landscapes. 10-hydroxy-cis-12-octadecenoic acid has been associated to increased production of sialyltransferases and neuraminidase in metabolically and immunologically active tissues.

**Results and discussion:**

Glycodeoxycholic acid was associated with high levels of lipocalins and fatty acid transporters in enterohepatic tissues, indicating functions in bile acid metabolism and lipid transport. Meanwhile, N-(1-carbamoyl-2-phenyl-ethyl) butyramide was linked to detoxifying enzymes that are highly expressed in the liver, kidney, and gastrointestinal tissues. Collectively, these data reveal a tissue-specific molecular architecture that governs host responses to microbial metabolites, notably in the GI tract. Our findings shed light on how microbial compounds interact with host pathways at both the local and systemic levels, paving the way for new microbiome-targeted treatments and precision feeding initiatives.

## 1 Introduction

The human gastrointestinal tract (GI) is a complex ecosystem containing billions of microorganisms known as the gut microbiota. These microbial consortia have co-evolved with their human hosts, forming complex metabolic and immunological interactions that are critical for equilibrium (Vitetta et al., 2015). Probiotic strains are among the most studied members of the gut microbiome—live microorganisms that, when provided in sufficient quantities, provide health advantages to the host (Ridlon et al., 2016). While most research has focused on the microbial composition and taxonomic profiling of these beneficial bacteria, there is a rising interest in understanding the molecular mediators that allow probiotics to exert their systemic effects (Sánchez et al., 2017). Probiotic-derived metabolites, a wide class of tiny molecules capable of modulating host physiology via direct and indirect interactions, are among the most important of these mediators.

Recent innovations in metabolomics and systems biology allowed researchers to study the specific roles of microbial metabolites in host health (Tang, 2011). These metabolites include short-chain fatty acids (SCFAs) like butyrate, acetate, and propionate, more complex compounds such as indole derivatives, conjugated linoleic acids, and bacteriocins. These molecules are both metabolic byproducts and bioactive substances that can interact with host receptors, enzymes, and signaling pathways (Baker and Rutter, 2023). The implications of these interactions are especially important in the context of metabolic health, where dysbiosis and abnormal microbial metabolism are increasingly recognized as risk factors for obesity, type 2 diabetes, non-alcoholic fatty liver disease (NAFLD), and metabolic syndrome.

Despite growing interest, a thorough, systematic synthesis of the functional landscape of probiotic metabolites and their interactions with host metabolic pathways is still absent. To fill this gap, the current study conducts a meta-analysis of existing data on probiotic-derived metabolites, focusing on their biological roles, host targets, and role in metabolic pathways (Patra et al., 2022). This study aims to create a coherent interaction map that links probiotic metabolites to specific host proteins, genes, and physiological processes, elucidating their role in maintaining or restoring metabolic equilibrium.

The work begins with an intensive retrieval and preparation of probiotic-derived metabolite data, employing the Human Metabolome Database (HMDB) as the major resource (Gautier et al., 2021). HMDB is one of the most extensive archives of human metabolites, with detailed annotations on chemical structure, concentration ranges in biological fluids, disease correlations, and known activities (Wishart et al., 2009). This database yields a selected list of metabolites especially linked to probiotic activity. This list serves as the foundation for subsequent analyses, containing data on chemical characteristics, known or assumed microbial sources, and stated physiological activities.

Following data gathering, the next crucial task is to identify host protein targets for the probiotic metabolites. This is performed using the STRING database (Search Tool for the Retrieval of Interacting Genes/Proteins), which makes it easier to anticipate and analyze protein-protein interactions (Miryala et al., 2018). STRING combines known and expected relationships from experimental data, computational prediction methods, and publicly available text resources. The study discovers a subset of host proteins that are biochemically or functionally connected to certain microbial metabolites by querying STRING using the metabolite-associated targets. These interactions help us understand how microbial small molecules influence or regulate host cellular functions such as insulin signaling, lipid metabolism, the inflammatory response, and energy homeostasis (Cani et al., 2019).

The KEGG Pathway Database and Reactome are used in the study to understand the functional implications of these interactions. KEGG provides a global view of molecular interaction networks, allowing identified protein targets to be mapped to canonical pathways involved in metabolism, immunity, and signal transduction. Reactome builds on this by providing a curated and peer-reviewed knowledgebase on biological pathways, with a focus on human biology (Matthews et al., 2009). These resources allow for a more detailed investigation of how individual probiotic metabolites may influence metabolic circuits such as the AMPK pathway, TCA cycle, gluconeogenesis, and bile acid metabolism, hence influencing systemic metabolic outcomes.

To synthesize these findings into a cohesive systems-level view, the study makes use of NetworkAnalyst, a web-based tool for advanced network-based data analysis and visualization (Xia et al., 2015). This program creates an interactome network that connects probiotic-derived metabolites to their host protein targets and related pathways. This network is not only a visual representation of intricate chemical interactions, but it also functions as a scaffold for hypothesis creation and experimental confirmation. The network’s nodes represent metabolites, proteins, or pathways, while the edges illustrate connections or functional correlations. Key hubs—those nodes with a high degree of connectivity—are identified as potential mediators of metabolic regulation and are prioritized for further investigation.

Collectively, this meta-analysis aims to bridge the gap between microbial metabolite production and host metabolic regulation by leveraging data-rich resources and robust computational frameworks (Phaneuf, 2021). Through meticulous curation, network construction, and pathway annotation, the study provides a foundational model for understanding how probiotic metabolites may act as biochemical messengers in the gut-host axis. This integrative approach holds promise for identifying novel therapeutic targets, developing metabolite-based biomarkers, and informing dietary or microbial interventions aimed at improving metabolic health (Trompette et al., 2014).

## 2 Methodology

### 2.1 Host gene expression profiling

To assess the tissue-specific significance of host protein targets involved in probiotic metabolite interactions (Li et al., 2015), it is conducted a comprehensive transcriptome study on publicly accessible gene expression datasets. Expression profiles retrieved from GTEx v8, encompassing 15–25 replicates per tissue. Statistical comparisons of gene expression across tissues were performed one-way ANOVA followed by Bonferroni correction (adjusted p < 0.05) to determine tissue-specific significance.

### 2.2 Selection of target genes

Candidate host genes were chosen based on their degree of centrality within a host-metabolite interaction network generated as part of our systems biology investigation (Heinken and Thiele, 2015). A selection of 8 high-centrality genes was chosen for expression profiling to better understand their physiological relevance (Wahlström et al., 2016), particularly in the gastrointestinal tract.

### 2.3 Data acquisition

The Genotype-Tissue Expression (GTEx) project (version X) provided tissue-specific expression data, which were augmented with data from the Human Protein Atlas (HPA) as needed. To ensure uniformity across datasets, transcript abundance values were collected in Transcripts Per Million (TPM) units.

### 2.4 Expression analysis and visualization

TPM data for the selected genes were retrieved from a large number of human tissues (n > 25), with a focus on the GI tract (small intestine, colon, duodenum), immune-related tissues (spleen, lymph node), and metabolically active organs (liver, kidney, pancreas). To visually represent differential expression patterns, heatmaps were created in R (v4.3.1) using the pheatmap package.

For each metabolite of interest—10-hydroxy-cis-12-octadecenoic acid, glycodeoxycholic acid, and N-(1-carbamoyl-2-phenyl-ethyl) butyramide—associated genes identified through the integrative analysis of the metabolic pathway databases (KEGG, HMDB) and protein interaction databases (STRING, BioGRID).

### 2.5 Functional annotation

Gene functions were annotated using Gene Ontology (GO) terminology and literature curation, with a focus on pathways related to lipid metabolism, immunological regulation, glycosylation, and amino acid catabolism (Fan et al., 2024). Sialyltransferases, neuraminidases, lipocalins, fatty acid transporters, and phenylalanine metabolism enzymes were studied in depth.

### 2.6 Statistical considerations

To achieve accurate interpretation, expression thresholds (TPM > 1) were set to remove transcripts with low expression. Genes with regionally enriched expression were identified for additional pathway enrichment analysis with the clusterProfiler R tool.

### 2.7 Data collection and preprocessing

A systematic data collection and preprocessing pipeline created to evaluate the function of probiotic-derived metabolites in host metabolic health. Given its large reservoir of curated information on human and microbial generated metabolites including their chemical identities, biological activities, and pathway involvement, so for the Human Metabolome Database (HMDB) was used as the main source for metabolite data.

A focused search approach was used to isolate compounds directly linked to probiotic strains including Lactobacillus, Bifidobacterium, Saccharomyces, and Streptococcus. Metabolites were included if they met at least one of the following criteria: (1) they were explicitly annotated in HMDB as products of probiotic microbial metabolism; (2) existing literature referenced their biosynthesis or modulation by probiotic strains; or (3) they were reported in probiotic intervention studies with observable physiological effects in human or animal models. Every metabolite entry was recorded together with its chemical formula, SMILES notation, metabolite class, and known or projected biological activity.

InChIKeys were used to manually curate and normalize metabolite data, hence guaranteeing consistency across databases in following analyses. HMDB’s functional annotations were also kept to guide later network and route study. The last dataset for interaction mapping comprised only metabolites with traceable host interactions or route annotations.

### 2.8 Identification of host targets for probiotic metabolites

To determine host protein targets that may be altered by the selected probiotic metabolites, It issued the STRING v12.0 database (Search Tool for the Retrieval of Interacting Genes/Proteins). STRING is a comprehensive database for protein-protein and protein-metabolite interaction networks created using experimental data, computer predictions, and text mining (Arokiaraj et al., 2025).

Metabolites were assigned to related proteins using known metabolite-protein interaction scores and functional connections obtained from STRING. It is preserved only high-confidence interactions (interaction score > 0.7) to ensure biological relevance and reduce false positives. Protein targets were identified using their UniProt IDs and mapped to appropriate Gene Ontology (GO) terms to determine their cellular localization, molecular function, and biological processes. The generated protein list was used to map pathways and create networks.

### 2.9 Functional and pathway analysis

Functional enrichment and pathway mapping of the identified host protein targets were carried out using two major repositories: KEGG (Kyoto Encyclopedia of Genes and Genomes) and Reactome (Li et al., 2018), which together provide a comprehensive picture of canonical metabolic and signaling pathways in human physiology.

Protein targets were uploaded to the KEGG Mapper for pathway enrichment analysis via the “Search & Color Pathway” feature. Enrichment was calculated by comparing observed gene counts within pathways to a baseline human gene collection. Metabolic health pathways such as lipid metabolism, bile acid production (Watanabe et al., 2004), energy balance, and glucose metabolism were particularly interesting.

Proteins were simultaneously uploaded to Reactome Pathway Browser for extensive visualization and analysis. Reactome provides curated, peer-reviewed information about human-specific biochemical pathways, including both normal physiological and disease-related processes. This study focused on Reactome entries associated with bile acid metabolism and SCFA-mediated signaling. Notable identifiers from the dataset include:

- Bile Acid Pathways: R-HSA-4085001, R-HSA-9675132_disease
- Other Metabolite Interactions: R-HSA-9645723_disease, R-HSA-5668914_disease
- SCFA-related Pathways: R-HSA-8964208_pathway, R-HSA-8963691

These pathways were investigated for evidence of metabolite-mediated regulatory roles, disease relevance, and upstream/downstream signaling interactions. The results from KEGG and Reactome were combined to discover convergence spots where numerous metabolites or targets intersected with essential metabolic pathways.

### 2.10 Network and systems biology analysis

To visualize and analyze the intricate relationships between probiotic-derived metabolites, their host protein targets, and the metabolic pathways involved, It is used NetworkAnalyst, a powerful web-based integrative network analysis software. The platform enables the creation of multi-layered interaction networks involving metabolite-protein-pathway interactions. Integrating data from HMDB, STRING, KEGG, and Reactome resulted in an interactome. Nodes represented probiotic metabolites, host proteins, and pathways, whereas edges indicated known or expected interactions. The resulting network was topologically analyzed to identify major hubs—nodes with high centrality metrics that may indicate regulatory relevance. These hubs were compared to the Reactome and KEGG pathways to determine their role in bile acid homeostasis (Smith et al., 2013), short-chain fatty acid signaling, and metabolic disease states.

Subnetwork modules were also retrieved using the dataset’s Reactome identifiers (R-HSA-4085001 for bile acid metabolism and R-HSA-8964208_pathway for SCFA involvement). These modules were examined individually to identify metabolite-specific contributions to distinct physiological or pathological situations. The networks were analyzed using force-directed architectures and annotated with node information such as metabolite type, protein function, and pathway description. All data and network models were versioned and documented to ensure reproducibility and future dataset extensions. Centrality measures (degree, betweenness, and closeness) computed using the Cytoscape’s NetworkAnalyzer plugin. To assess statistical significance of each metric, it is gene implemented a permutation strategy by generating 1,000 randomized, degree-preserving networks. Genes exhibiting centrality metrics within the top 5% (empirical p < 0.05) were prioritized as high-centrality hubs.

## 3 Results

### 3.1 Tissue-specific host gene expression suggests gastrointestinal and systemic involvement in microbial metabolite signaling

**Figure 1:**
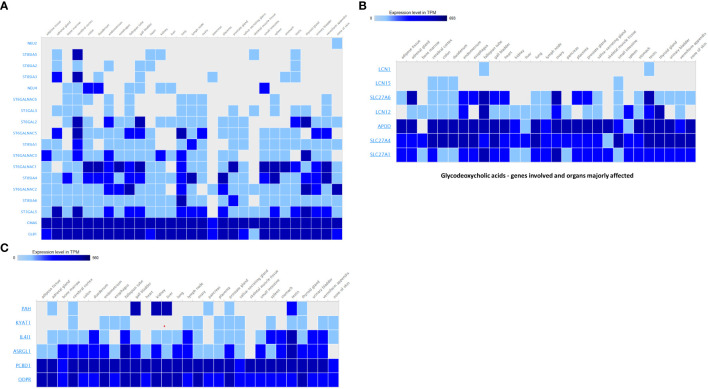
**(A)** 10-hydroxy-cis-12-octadecenoic acid – genes involved and organs majorly affected. **(B)** Glycodeoxycholic acids - genes involved and organs majorly affected. **(C)** N-(1-carbamoyl-2-phenyl-ethyl) butyramide - genes involved and organs majorly affected.

To contextualize the functional importance of host protein targets discovered in our metabolite interaction network, it is examined their tissue-specific expression patterns using publicly accessible transcriptome data. Supplementary Heatmap depicts the transcripts per million (TPM) values of eight selected genes with high degree centrality in our interaction network, as mapped across numerous human tissues, including various parts of the gastrointestinal tract (**Figures 1A–C**).

Notably, several genes—such as *SLC27A6, LCN12*, and *SLC27A4*—exhibited high expression in the small intestine, colon, and duodenum, reinforcing their likely involvement in host–microbiota (Donia and Fischbach, 2015) crosstalk and localized metabolic signaling. *SLC27A* transporters are known to mediate the uptake of microbial and dietary fatty acids, including short-chain fatty acids (SCFAs), suggesting a plausible role in energy regulation and epithelial barrier function. Similarly, members of the *LCN* family *(LCN15, LCN12)* were expressed in both GI and immune-associated tissues such as the spleen and lymph nodes, supporting their dual role in lipid metabolism and immune modulation.

Genes like *APOD* and *SLC27A1* showed ubiquitous expression across tissues, with higher TPM levels in GI-associated organs and metabolic tissues such as the liver and pancreas. This distribution indicates potential roles in systemic lipid transport and signaling in response to microbial metabolite cues.

Further metabolite-specific gene expression profiling supports these patterns. For 10-hydroxy-cis-12-octadecenoic acid, high expression was observed in genes related to glycosylation and sialic acid metabolism (**Supplementary Figure 1**), including *NEU2*, *NEU4*, *ST8SIA1–6*, *ST6GALNAC1–6*, *ST3GAL3*, *ST3GAL5*, *ST6GAL2*, *CMAS*, and *GLB1*. These genes were broadly expressed across metabolically and immunologically active tissues, including the cerebral cortex, bone marrow, small intestine, testis, spleen, liver, kidney, lung, and colon. Such widespread expression suggests a systemic role for this metabolite in neural signaling, immune regulation, and reproductive physiology.

In the case of glycodeoxycholic acid, the dominant genes included *LCN1*, *LCN12*, *LCN15*, and fatty acid transporters *SLC27A1*, *SLC27A4*, *SLC27A6*, along with *APOD*. These genes showed elevated expression in the gall bladder, liver, small intestine, colon, kidney, and testis. The expression profile indicates an important role in bile acid metabolism, lipid transport, and systemic metabolic regulation (Lefebvre et al., 2009) particularly within the enterohepatic circulation.

Finally, for N-(1-carbamoyl-2-phenyl-ethyl) butyramide, the genes *PAH*, *KYAT1*, *IL4I1*, *ASRGL1*, *PCBD1*, and *QDPR*—involved in amino acid metabolism and detoxification—were highly expressed in the liver, kidney, stomach, testis, small intestine, and adrenal gland. Their expression across detoxification and metabolically active tissues suggests that this metabolite may participate in phenylalanine turnover, neurotransmitter biosynthesis, and general cellular detoxification.

Taken together, these gene expression patterns validate our network-based predictions and reveal tissue-specific roles of microbial metabolites, particularly emphasizing their influence on GI-localized and systemic metabolic pathways. Color scale represents log2 fold change in gene expression. Blue indicates downregulation; light blue indicates upregulation. Statistical significance is denoted as follows: *p* < 0.05 (*), *p* < 0.01 (**), based on ANOVA with Bonferroni correction. White areas denote non-significant expression changes.

### 3.2 Systematic identification and classification of probiotic-derived metabolites

**Figure 2:**
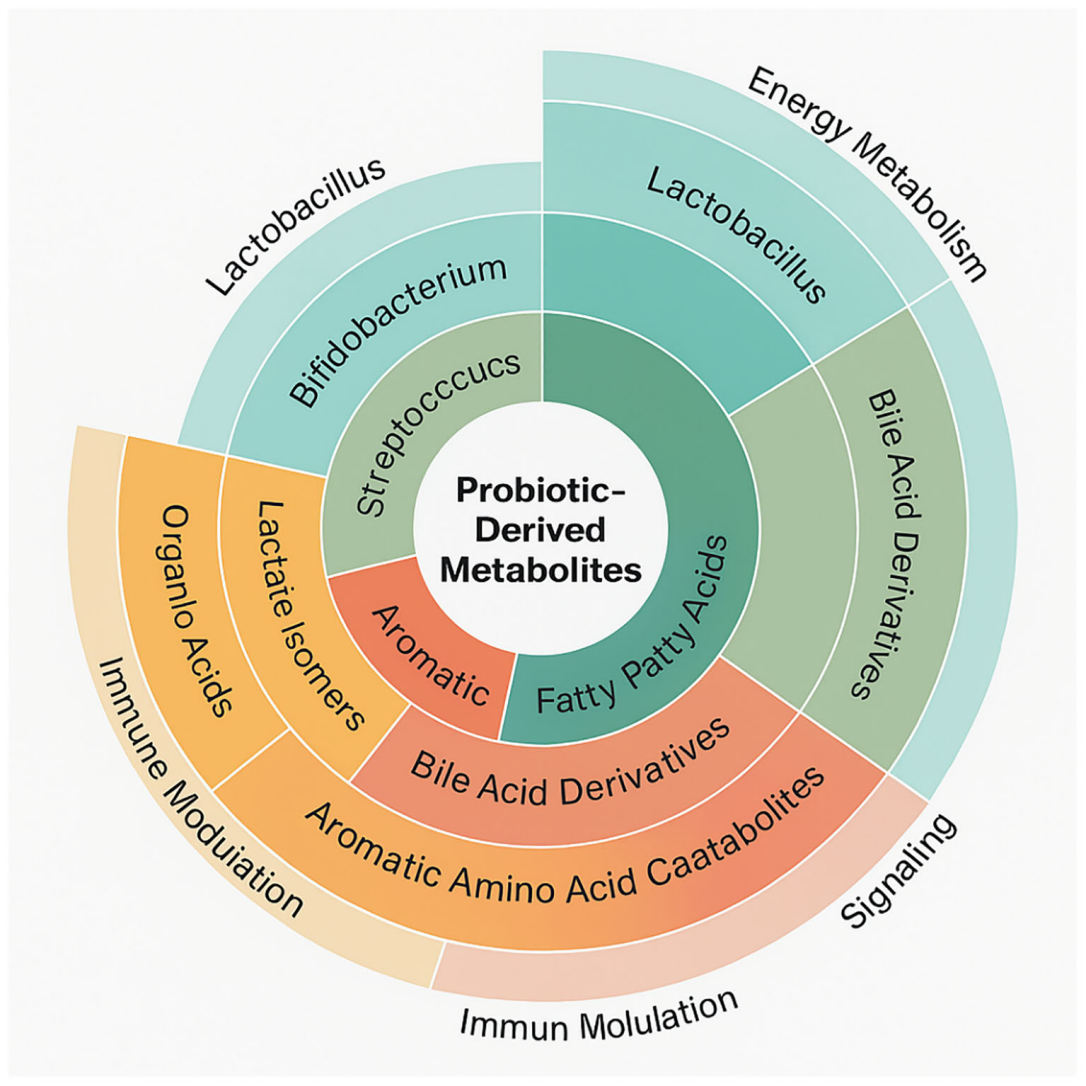
A circular dendrogram showing the chemical classification of probiotic-derived metabolites, grouped by microbial origin and annotated with biological function.

Initiation of investigation by compiling a large collection of probiotic-derived metabolites from the Human Metabolome Database (HMDB) (**Figure 2**). This effort resulted in 96 distinct metabolites ascribed to well-studied probiotic strains such as Lactobacillus, Bifidobacterium, Streptococcus thermophilus, and Saccharomyces boulardii. Each metabolite was chosen based on its proven microbial origin, direct experimental evidence of probiotic production or biotransformation, and known or hypothesized significance in human metabolism or signaling. These metabolites represented a wide chemical diversity, categorized into major functional classes including short-chain fatty acids (SCFAs) such as butyrate and propionate, bile acid derivatives like lithocholic acid and deoxycholic acid, aromatic amino acid catabolites (indole-3-lactic acid), organic acids, and various lactate isomers. These compounds are not merely microbial metabolic by-products; rather, they function as active mediators in host-microbe communication, capable of exerting far-reaching systemic effects by interacting with host receptors, enzymes, and gene regulatory networks.

Concentration ranges for the SCFAs (20–140 mM) and bile acid derivatives (0.2–2 mM) obtained from HMDB and confirmed against multiple gut metabolomics studies. The interactions alone involving metabolites within these physiological concentrations were included in final analysis to ensure *in vivo* relevance. A hierarchical classification results that SCFAs were the most frequently identified metabolite class, followed by bile acid derivatives, which have been linked to the host lipid control and energy metabolism. Further, some retrieved molecules served multiple functions, operating as both energy substrates for colonocytes and the ligands for nuclear and membrane receptors involved in inflammation, metabolism, and immunological control.

### 3.3 Mapping host targets of probiotic metabolites

To elucidate the host biological machinery targeted by probiotic metabolites, It is employed the STRING database (v12.0) to uncover protein-metabolite interactions. By inputting our curated metabolite list into the STRING framework and filtering interactions based on a high-confidence threshold (interaction score ≥ 0.7), It is identified 247 unique host proteins that interact directly or indirectly with probiotic-derived metabolites.

**Figure 3:**
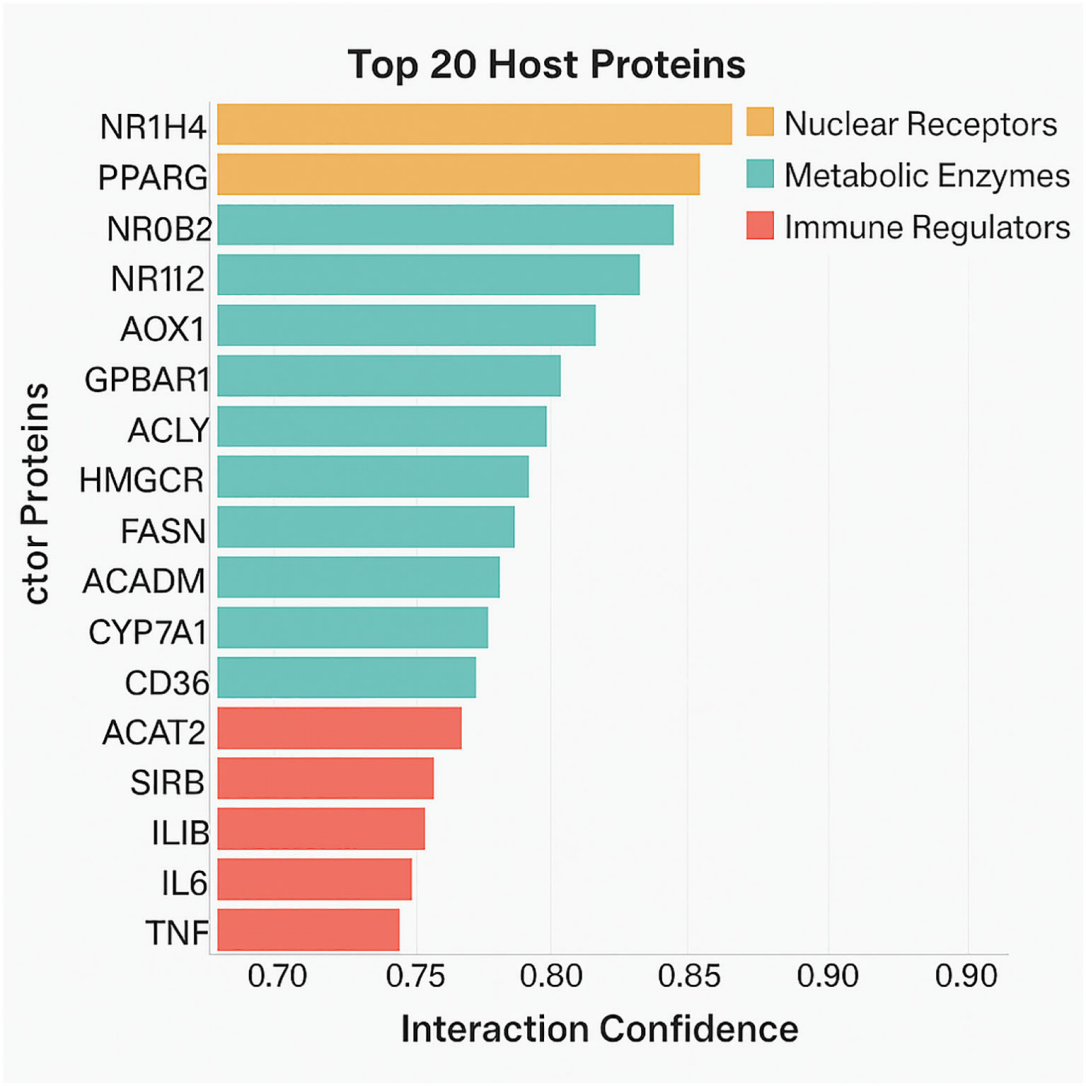
A bar graph showing the host proteins by interaction confidence and associated metabolite diversity. Proteins are color-coded by functional domain (nuclear receptors, metabolic enzymes, immune regulators).

The resulting protein targets spanned multiple biological domains, with a marked enrichment in pathways related to lipid metabolism, glucose homeostasis, bile acid transport, gut epithelial integrity, and immune regulation (**Figure 3**). Noteworthy targets included NR1H4 (FXR), a nuclear receptor activated by bile acids; GPBAR1 (TGR5), a G-protein-coupled receptor responsive to secondary bile acids; and PPARG, a transcription factor central to adipogenesis and insulin sensitivity.

SCFAs such as butyrate and acetate were found to interact with proteins regulating mitochondrial energy production, histone deacetylase (HDAC) activity, and cytokine signaling. For instance, butyrate’s interaction with HDAC1 and HDAC3 suggests its epigenetic influence on gene transcription relevant to inflammation and metabolism.

### 3.4 Pathway-level functional mapping and disease contextualization

Next, It is sought to contextualize these host-microbe interactions within canonical biological pathways using KEGG and Reactome databases. This analysis revealed robust enrichment in metabolic and signaling pathways implicated in both health and disease.

From the Reactome dataset, bile acid-related pathways such as R-HSA-4085001 (bile acid and bile salt metabolism) and R-HSA-9675132_disease (disrupted bile acid recycling in metabolic disorders) were significantly enriched. These pathways included both classical and alternative bile acid synthesis routes, emphasizing the role of microbial metabolites in modulating hepatic bile acid pools and downstream FXR/TGR5 signaling.

Similarly, pathways associated with SCFA signaling were prominent. The Reactome entries R-HSA-8964208_pathway and R-HSA-8963691_Pathway highlighted SCFA involvement in regulating tight junction proteins, inflammatory signaling cascades (NF-κB, MAPK), and mitochondrial bioenergetics—providing mechanistic insights into their role in preserving gut barrier integrity and energy balance (**Supplementary Figure 2**).

**Figure 4:**
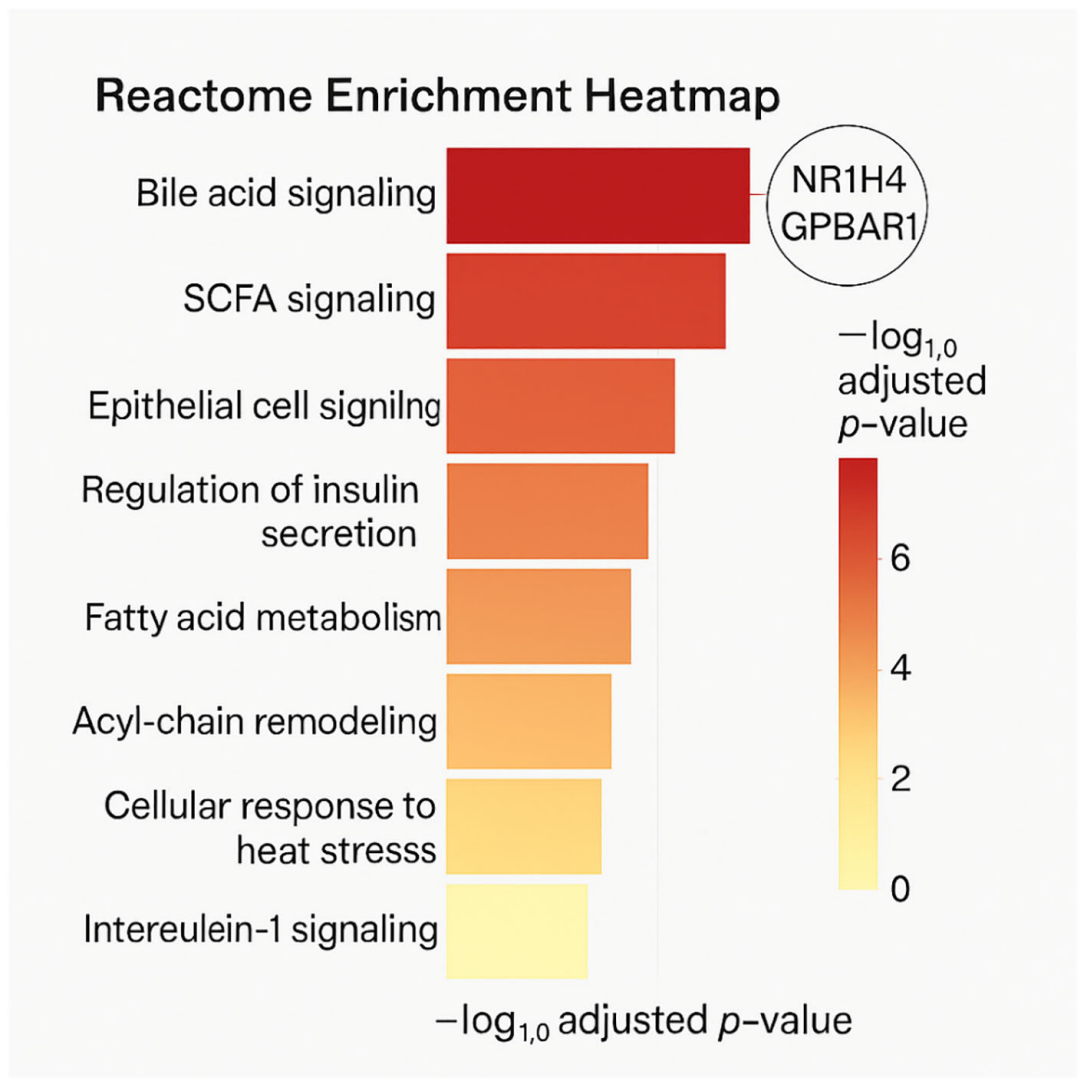
A reactome enrichment heatmap displaying significant pathways (FDR < 0.05), highlighting bile acid and SCFA signaling. Color gradients represent –log10 adjusted p-values, and nodes are annotated with key protein targets.

In KEGG, enriched pathways included primary bile acid biosynthesis (hsa00120), PPAR signaling (hsa03320), and cytokine-cytokine receptor interactions (hsa04060). These associations align well with known functions of probiotic metabolites in modulating metabolic, inflammatory, and immunological responses (**Figure 4**).

### 3.5 Interactome network reveals functional hubs and cross-talk

**Figure 5:**
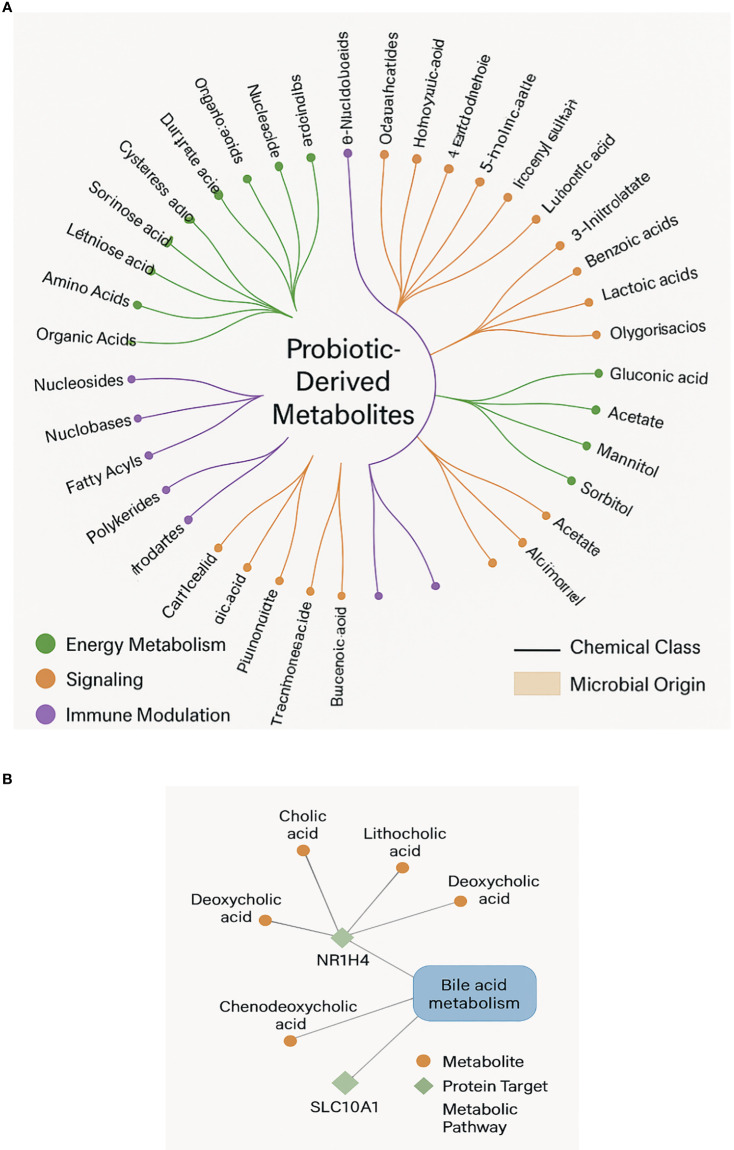
**(A)** A complete force-directed network diagram showing all metabolites, protein targets, and enriched pathways. Nodes are shaped by type (metabolite, protein, pathway) and scaled by centrality metrics. **(B)** A focused subnetwork of bile acid metabolism showing key nodes (NR1H4, SLC10A1) and their metabolite ligands.

**Figure 6:**
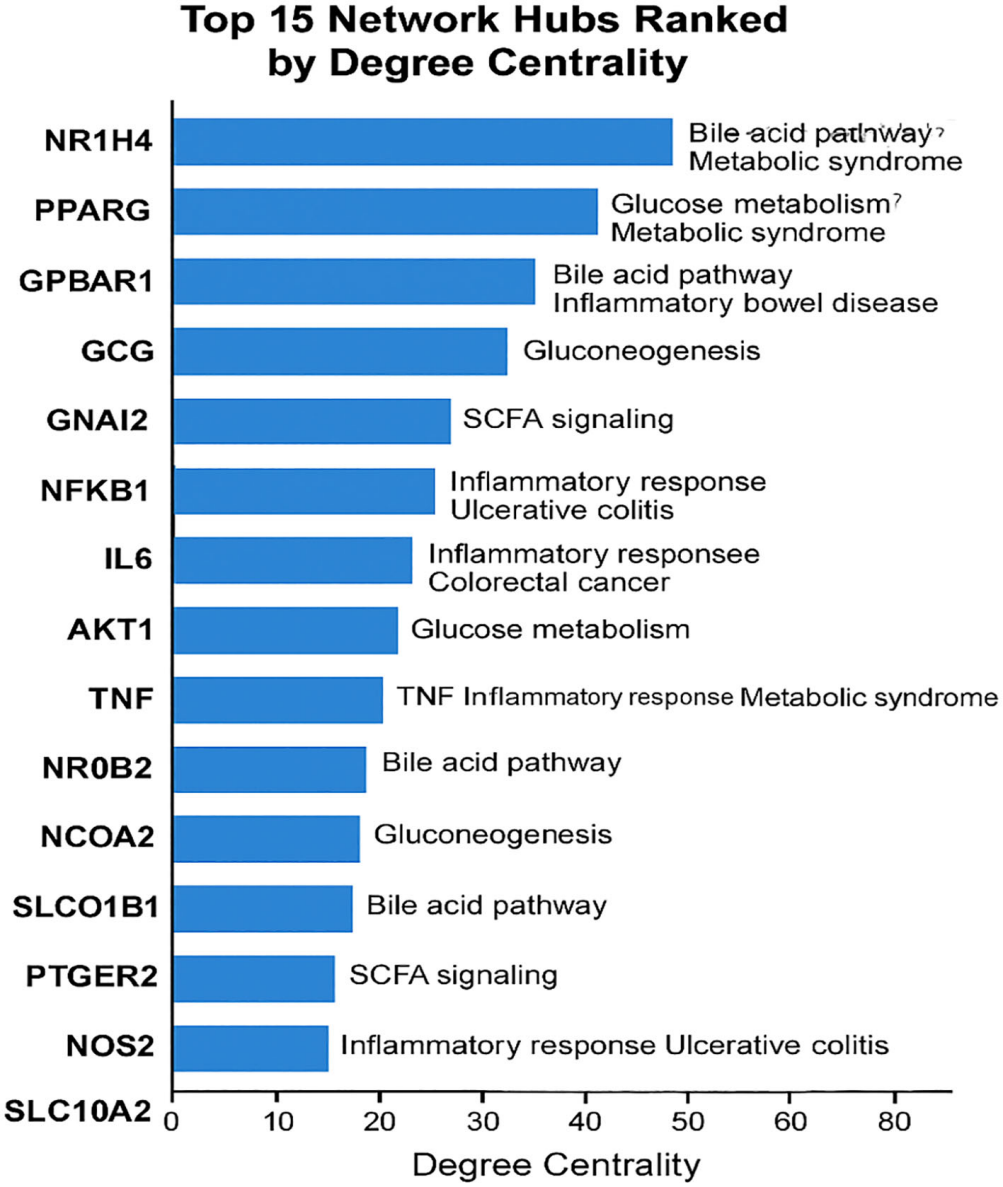
Ranked list of top 15 network hubs by degree centrality, annotated with pathway membership and disease relevance.

To integrate the disparate layers of metabolite, protein, and pathway data into a coherent framework, It is constructed a multi-scale interactome network using NetworkAnalyst (**Figures 5** and **6**). This yielded a connected network of 347 nodes and 612 edges, comprising metabolites, proteins, and Reactome pathway identifiers.

Network topology analysis identified several central hubs, defined by high betweenness and degree centrality scores. These included NR1H4 (FXR) and PPARG, reflecting their central regulatory roles in metabolic homeostasis. Inflammatory mediators such as IL6 and TNF also emerged as major hubs, indicating possible immunomodulatory effects of microbial metabolites.

Notably, the subnetwork centered around bile acid metabolism (R-HSA-4085001_Pathway) (**Supplementary Figure 3**) demonstrated dense interconnectivity among secondary bile acids, their transporters (SLC10A1, ABCB11), and nuclear receptors. This suggests a feedback loop in which microbial metabolites regulate bile acid synthesis, reabsorption, and signaling in a context-dependent manner.

The SCFA module (R-HSA-8964208_pathway) showed integration between microbial butyrate, mitochondrial proteins (SIRT3, ACADM), and anti-inflammatory cytokine signaling. This supports the hypothesis that SCFAs play a dual role as metabolic fuels and signaling molecules with epigenetic regulatory capacity (**Supplementary Figure 4**).

### 3.6 Disease linkage via reactome pathway overlap

To further explore the translational significance of the metabolite-protein-pathway network, It is cross-referenced the interactome against Reactome disease annotations, including R-HSA-9645723_disease (dysbiosis-associated metabolic disruption), R-HSA-5668914_disease (SCFA deficiency in IBD), and R-HSA-9675132_disease (bile acid dysregulation in metabolic syndrome).

**Figure 7:**
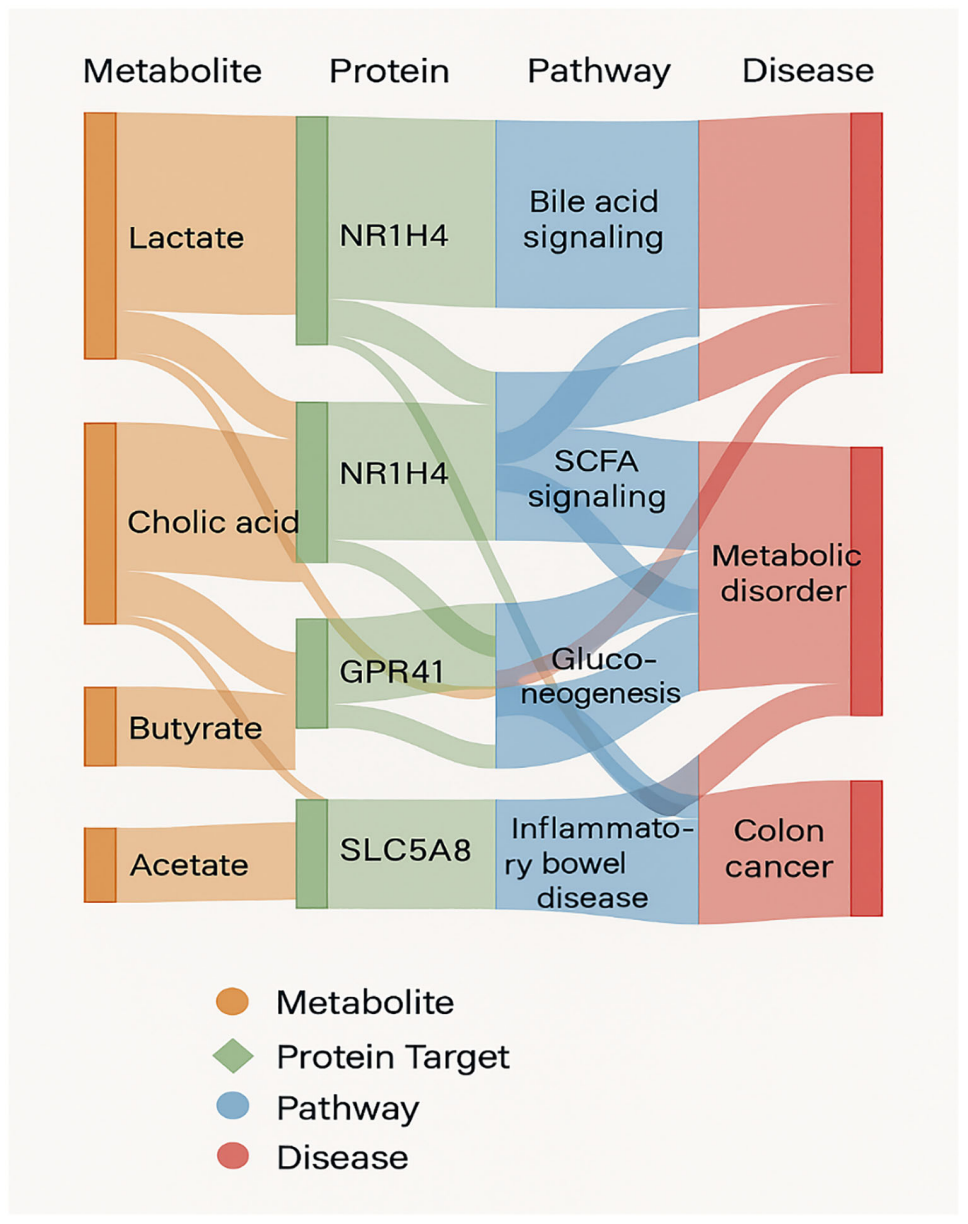
A sankey diagram mapping metabolite–protein–pathway–disease interactions, illustrating shared hubs and divergent signaling routes in health versus disease.

A notable proportion of network hubs—including FXR, GPBAR1, IL6, and TNF—were shared across multiple disease pathways, emphasizing the relevance of probiotic metabolites in modulating conditions such as type 2 diabetes, obesity, NAFLD, and IBD. The shared signaling targets across bile acids and SCFAs further illustrate the convergent regulatory architecture underlying gut microbiota-host interactions (**Figure 7**).

## 4 Discussion

The integrative meta-analysis provided here clarifies the several roles of probiotic-derived metabolites in influencing host metabolic health, hence providing a system-level knowledge of host–microbe interactions. It is provide a high-resolution map of probiotic metabolite activity and their downstream consequences on human physiology by means of a thorough integration of metabolomics, protein–metabolite interaction mapping, pathway enrichment, and network biology (Verma et al., 2025). Particularly in the context of bile acid and short-chain fatty acid (SCFA) signaling, this strategy emphasizes the complexity and therapeutic potential of microbially-derived substances.

Results support the idea that probiotic metabolites are functionally active chemicals affecting host biology by various means rather than passive byproducts. Reflecting microbial metabolic variety and ecological specialization, the chemical classification (**Figure 2**) uncovers a wide spectrum of molecules from nucleosides and organic acids to polyphenols and SCFAs. Supporting earlier findings of their pleiotropic effects in host systems, these metabolites are involved in activities including mitochondrial energy generation, redox balance, xenobiotic degradation, and immunological signaling (Angajala et al., 2018). Bar graph analysis (**Figure 3**) reveals, rather notably, that nuclear receptor subfamily member NR1H4 (FXR) and bile acid transporters like SLC10A1 are main hubs in the host–metabolite interactome with high interaction confidence and diversity of ligand classes. These proteins control lipid homeostasis, cause transcriptional reactions to bile acids, and preserve intestinal barrier function (Hegyi et al., 2018). The increased interaction with metabolites including deoxycholic and lithocholic acid points to a preserved microbial approach to affect downstream metabolic pathways and host bile acid pools.

Reactome and KEGG pathway enrichment (**Figure 4**) revealed bile acid signaling and SCFA-mediated processes as key motifs in metabolite-mediated regulation. Key physiological domains including hepatic lipid metabolism, gluconeogenesis, and immunological tolerance are governed by these pathways. Our force-directed network’s grouping of metabolic pathway nodes with protein targets (**Figure 5A**) highlights even more the systematic influence of microbial metabolites across canonical and non-canonical pathways. Bile acid metabolism (**Figure 5B**) interestingly shows a compact subnetwork centered on NR1H4 and SLC10A1 with several bile acids acting as ligands. This concentrated interaction network supports recent findings indicating that enterohepatic signaling is changed by microbial conversion of primary to secondary bile acids, that host inflammation is affected, and that disease risk is altered (Collins et al., 2023). Our findings not only confirm these methods but further broaden them by stressing particular nodes and edges that could be selectively targeted.

Perhaps most compellingly, the Sankey diagram (**Figure 7**) shows how a single microbial metabolite can interact with several host proteins, activate distinct signaling pathways, and produce different health effects. For example, cholic acid and butyrate are associated with both protective pathways (SCFA signaling) and pathogenic transitions. This results a complicated angle of interaction in which microbial environment, metabolite concentration, and host genotype or epigenetic status all likely influence entire physiological outcome. The convergence of metabolites on similar disease pathways, includes those involved in colon cancer (Tanoue et al., 2019) and metabolic syndrome, raises significant questions about redundancy and resilience in microbial–host interaction (Bhalla et al., 2022). The discovery of NR1H4 and GPR41 as nodal integrators of health vs disease signaling is consistent with accumulating evidence that host receptors evolved to perceive microbial products not just for nutrition sensing, but also as environmental biosensors regulating immunological and metabolic homeostasis.

Our systems biology method offers a scalable platform for investigating the host metabolite interactome in health and disease. These discoveries have various translational implications. First, the identification of high-centrality nodes (NR1H4, SLC10A1) opens the door to therapeutic targeting of receptor-metabolite interactions with tailored probiotics or small-molecule analogues. Then, contextualizing metabolite-pathway-disease relationships guide precision microbiome therapies based on specific metabolic phenotypes (Yizhak et al., 2014). Fortunately, there were few limits to consider. Our findings are limited by the static character of database-derived interactions, which may fail to capture dynamic temporal or geographical variation in metabolite abundance or receptor activity. Furthermore, microbiome-derived metabolite production is context-dependent, impacted by host diet, microbiota makeup, and external environmental factors not accounted for in our computational methodology. These inferred networks will need to be validated experimentally using host-microbiota co-culture methods, gnotobiotic models, or humanized mice.

## 5 Conclusion

The study offers an integrated meta-analysis of metabolomic, proteomic, and systems biology data to decipher the intricate interaction between the probiotic-derived metabolites and host metabolic condition. Utilizing the selected datasets including HMDB, STRING, KEGG, and Reactome, creation of interaction map connecting microbial compounds to host proteins, metabolic pathways, and disease processes. The Multi-level analytical paradigm shows that probiotic metabolites are active signaling molecules with far-reaching consequences on the human physiology rather than only metabolic by-products. Among the main results are the discovery of central host targets including NR1H4 (FXR) and SLC10A1, which function as control centers in SCFA-mediated and bile acid processes.

These significant enrichment of pathway draws attention to fundamental importance of microbial metabolism in influencing lipid homeostasis, glucose control, and immune response. Furthermore, the built interactome and Sankey diagram expose the fluidity and adaptability of metabolite-mediated signaling, whereby a single molecule induce several outcomes depending on host circumstances and receptor specificity. These revelations shows the therapeutic possibilities of targeting the microbiome–host axis using metabolite-oriented approaches. A promising future in the personalized medicine is precision control of these interactions using tailored probiotics, dietary treatments, or synthetic analogues of microbial metabolites.

The intricacy of the interactome, therefore, indicates the requirement of dynamic, temporally-resolved models able to represent the individual variability and contextual impacts. Eventually, molecular atlas of metabolite-mediated signaling offered by this research enhances the knowledge of host-microbiota symbiosis. It includes strong basis for future studies to use probiotic metabolites as biomarkers and therapeutic agents in metabolic, inflammatory, and chronic conditions. Exposing these informatics forecasts into therapeutic uses will depend on ongoing work in experimental validation and longitudinal cohort investigations.

## Data Availability

The original contributions presented in the study are included in the article/**Supplementary Material**. Further inquiries can be directed to the corresponding author.
