# Supplementary material for: Meta-analysis of probiotics metabolites in gastrointestinal tract and metabolic health
Source: Front Cell Infect Microbiol. 2025 Jun 19;15:1619501. doi: 10.3389/fcimb.2025.1619501 (PMC12222185; doi:10.3389/fcimb.2025.1619501)

**Figure S1:** Sialic acid metabolism


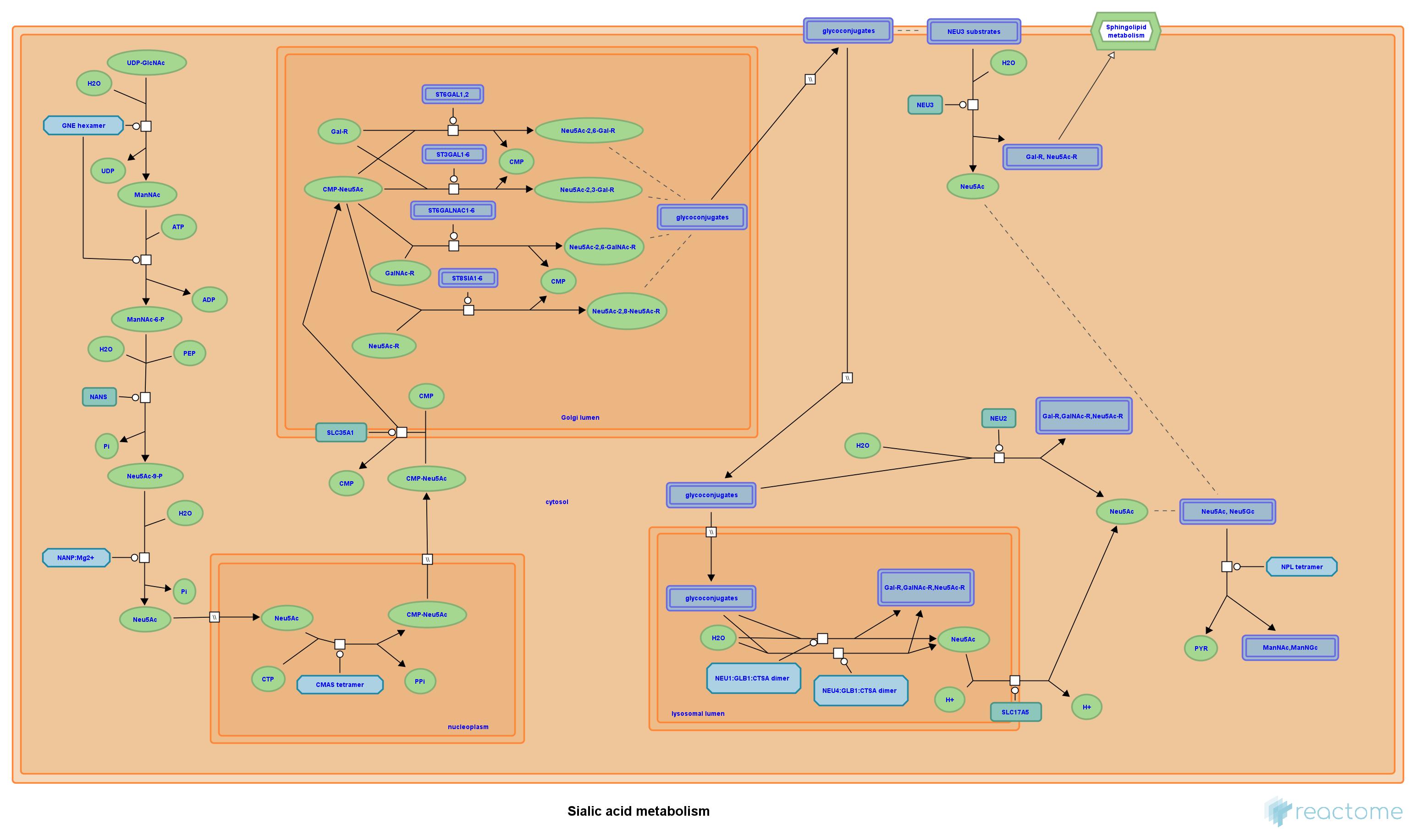


**Figure S2:** R-HSA-8963691_Pathway


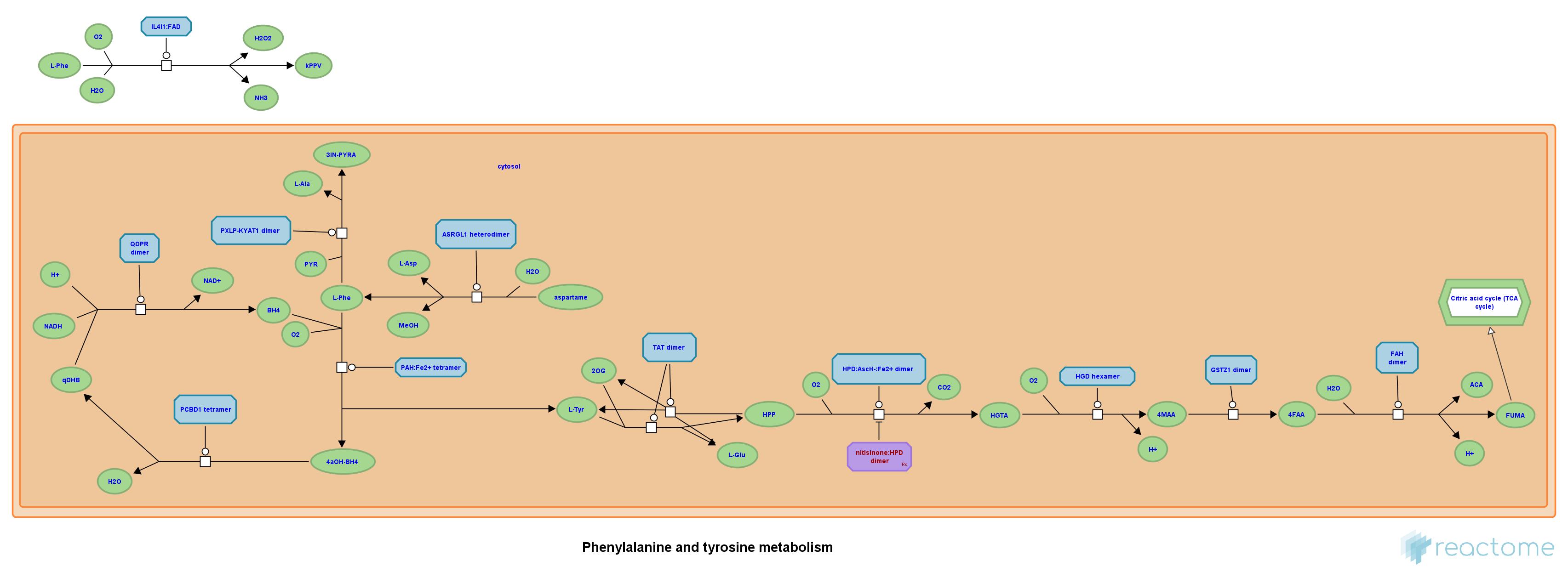


**Figure S3:** Bile acid metabolism (R-HSA-4085001_Pathway)


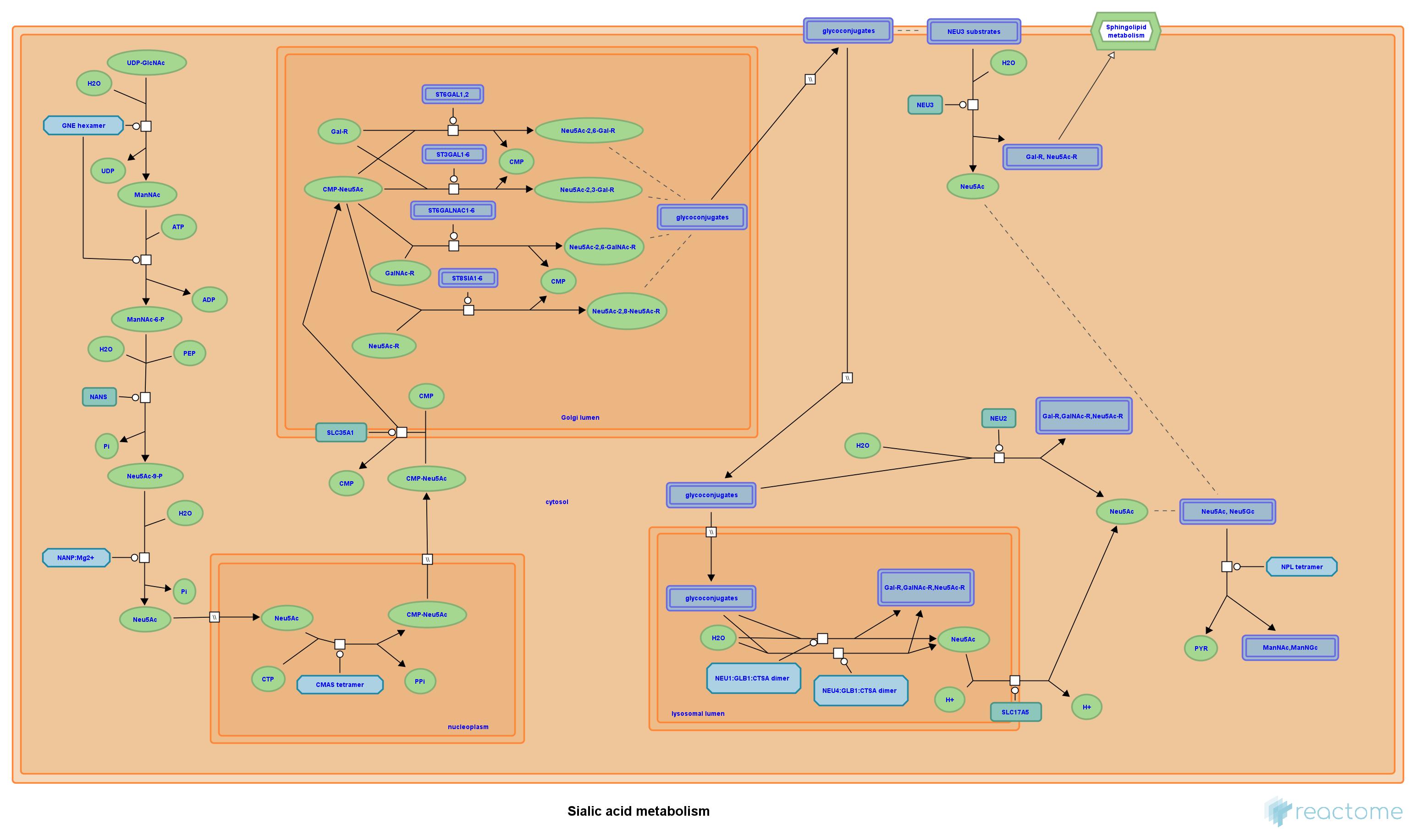


**Figure S4:** R-HSA-8964208_pathway


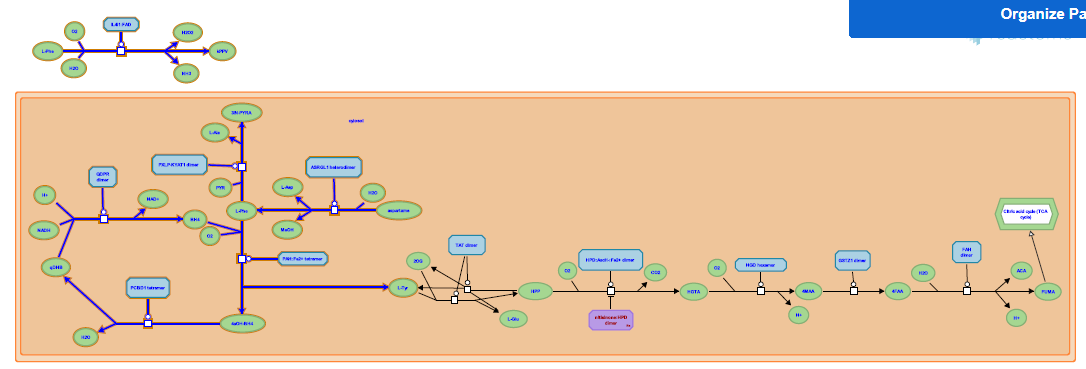

Supplement: Supplementary file 1 [file DataSheet1.docx]
